# The Impact of Provider’s Quality of Information System on User Satisfaction and Perceived Net Benefits in Malaysian Public Hospitals

**DOI:** 10.21315/mjms-12-2024-952

**Published:** 2025-04-30

**Authors:** Muhd Siv Azhar Merican Abdullah, Azimatun Noor Aizuddin, Mohd Rizal Abdul Manaf

**Affiliations:** 1 Department of Public Health Medicine, Faculty of Medicine, Universiti Kebangsaan Malaysia, Kuala Lumpur, Malaysia; 2 Ministry of Health Malaysia, Hospital Tengku Permaisuri Norashikin, Kajang, Selangor, Malaysia

**Keywords:** provider’s quality, information system, user satisfaction, perceived net benefits, public hospitals

## Abstract

**Background:**

Hospital Information Systems (HIS) are pivotal in enhancing decision-making, operational efficiency, and patient care. The quality of the provider-supported HIS, particularly in outsourced settings, critically influences user satisfaction and perceived net benefits. This study investigates the impact of provider quality, including system, service, and information quality, on user satisfaction and perceived net benefits in Malaysian public hospitals. Employing the DeLone and McLean Information System Success Model as a theoretical framework, this study also explores the mediating role of user satisfaction.

**Methods:**

Structural Equation Modelling using Analysis of Moment Structures (AMOS) was applied to data from 1,376 respondents across six hospitals.

**Results:**

The structural model revealed significant direct effects: system quality (*β* = 0.480, *P* < 0.001) and service quality (*β* = 0.438, *P* < 0.001) positively impacted user satisfaction, whereas information quality had no significant effect (*P* = 0.232). Service quality also significantly influenced perceived net benefits (*β* = 0.135, *P* < 0.001), as did user satisfaction (*β* = 0.342, *P* < 0.001). The indirect effects highlighted user satisfaction as a key mediator, with significant mediation observed for system quality (*β* = 0.164, *P* < 0.001) and service quality (*β* = 0.150, *P* < 0.001).

**Conclusion:**

This study highlights the critical mediating role of user satisfaction, providing deeper insights into user interactions in healthcare settings. In addition, it offers valuable contributions to understanding how system quality and service quality impact user satisfaction and perceived net benefits, along with practical recommendations for improving HIS.

## Introduction

Hospital Information Systems (HIS) play an essential role in enhancing decision-making, operational efficiency, and patient care. Globally, the quality of HIS determines its usability and effectiveness, making it a cornerstone of user satisfaction and overall system success. DeLone and McLean’s Information System Success Model (1) highlights system quality as a key determinant of information system success, emphasising its critical role in achieving intended outcomes.

In Malaysia, public hospitals rely heavily on HIS to manage resource constraints and improve service delivery. However, achieving seamless system integration and ensuring high-quality provider-supported systems remain challenging, particularly in outsourced environments. Local studies, such as those by Bakar et al. (2), have reported mixed outcomes, with partial failures resulting from integration issues and operational inefficiencies.

Despite its potential, HIS implementation often faces significant challenges globally and locally. Although some studies have acknowledged the importance of provider support in their frameworks (3–6), issues such as poor vendor performance, delayed implementations, and lack of vendor commitment continue to undermine system effectiveness and user satisfaction internationally (7, 8). Research indicates that only 33% of users are satisfied with outsourced systems (9), highlighting the critical need for reliable provider support. Similarly, in Malaysia, public hospitals encounter comparable challenges, including suboptimal HIS performance and poor system integration (2).

Although existing research acknowledges these challenges, there is limited understanding of how provider system quality impacts user satisfaction and perceived net benefits, especially in outsourced settings. Previous studies (7, 9) emphasise delays and provider-related issues but have not explored how these factors interact with satisfaction and benefit perception. This gap necessitates further investigation into the interplay between the provider’s quality, satisfaction, and perceived benefits, particularly in the context of Malaysian public hospitals.

User satisfaction mediates the relationship between system quality and perceived net benefits. High-quality systems that meet functional and operational expectations foster satisfaction, which, in turn, enhances the perception of benefits. For example, in a public hospital setting, a reliable HIS that minimises delays in accessing patient records not only supports clinical workflows but also reinforces user trust and satisfaction. Conversely, technically advanced systems may fail to deliver perceived benefits if users find them cumbersome or misaligned with their needs.

The primary objective of this study was to evaluate the impact of provider system quality on user satisfaction and perceived net benefits in Malaysia’s public hospitals. In addition, it examines the mediating role of user satisfaction, which links system quality to perceived benefits. The theoretical framework of this study is based on the DeLone and McLean Information System Success Model, which provides a comprehensive foundation for understanding how system quality drives user satisfaction and net benefits. In this research, system quality was tailored to include healthcare-specific attributes such as modularity and integration, ensuring relevance to the unique context of HIS in public hospitals.

This research explores these factors to offer evidence-based insights for policymakers and practitioners. For policymakers, it provides recommendations for optimising HIS outsourcing contracts and improving provider accountability. For healthcare practitioners, this study highlights strategies to enhance system quality and user satisfaction, ensuring better HIS integration and functionality. Academically, the research contributes to the broader literature on information systems in healthcare, addressing critical gaps in outsourcing challenges and their implications for HIS success.

**Figure 1:**
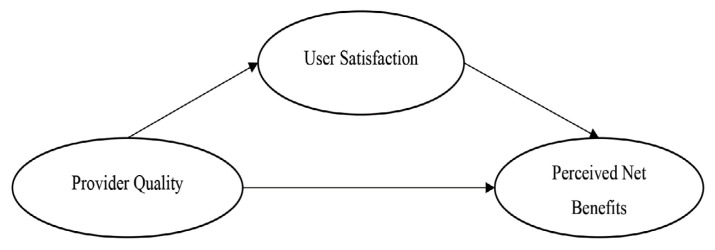
Conceptual framework

The theoretical foundation of this study is the DeLone and McLean Information System Success Model (1), which identifies system quality, information quality, and service quality of the provider as critical dimensions of information system success. In this study, the model is tailored to the healthcare context, focusing on the role of provider-supported system quality, which is defined by attributes such as modularity, reliability, and integration in influencing user satisfaction and perceived net benefits (Figure 1).

### Provider Quality

Service Quality refers to the technical support that end users receive from vendors and IT personnel and encompasses responsiveness, accuracy, reliability, technical competence, and empathy (10). In the context of HIS, this includes ensuring seamless technical assistance, robust system integration, and adequate user training to support clinical workflows. The SERVQUAL model (11) is often used to evaluate service quality by measuring the gap between user expectations and perceptions of service performance. A narrower gap indicates higher perceived service quality.

Provider quality encompasses three key dimensions: service quality, information quality, and system quality, which collectively determine the overall effectiveness of an HIS. Service Quality refers to the technical support that end users receive from vendors and IT personnel and encompasses responsiveness, accuracy, reliability, technical competence, and empathy (10). In the context of HIS, this includes ensuring seamless technical assistance, robust system integration, and adequate user training to support clinical workflows. The SERVQUAL model (11) is often used to evaluate service quality by measuring the gap between user expectations and perceptions of service performance. A narrower gap indicates higher perceived service quality. Service quality is particularly critical in outsourced HIS environments, where providers are responsible for system maintenance, user support, and troubleshooting (8, 9). Studies have demonstrated that poor vendor or provider performance and delayed implementations are common barriers to achieving optimal service quality in HIS (8, 9). In Malaysia, public hospitals face challenges such as limited provider accountability and integration issues, which compromise system quality and, consequently, user satisfaction (2).

Information quality refers to the accuracy, relevance, completeness, and timeliness of the data provided by the system. In HIS, high-quality information ensures that healthcare professionals have reliable data to make informed decisions, thereby improving patient care and operational efficiency (1). System quality refers to the technical performance and usability of the system itself. A high-quality system is one that operates efficiently, integrates seamlessly with other healthcare technologies, and is easy for users to navigate (1).

When the service, information, and system quality dimensions are managed effectively, they contribute to the overall provider quality, which plays a crucial role in determining user satisfaction and the perceived net benefits of the healthcare information system (8, 9). Empirical studies have demonstrated that provider system quality significantly affects user trust, system adoption, and overall satisfaction. For instance, a reliable HIS that supports timely access to patient records fosters trust and encourages user engagement. Conversely, systems with frequent technical failures or inadequate support reduce satisfaction and hinder perceived benefits (7, 8).

Empirical studies have demonstrated that provider system quality significantly impacts user trust, system adoption, and overall satisfaction. For instance, a reliable HIS that supports timely access to patient records fosters trust and encourages user engagement. Conversely, systems with frequent technical failures or inadequate support reduce satisfaction and hinder perceived benefits (7, 8).

### User Satisfaction

User satisfaction is a critical determinant of HIS success, reflecting user perceptions of the system’s functionality, usability, and support quality. According to the DeLone and McLean Information System Success Model (1), satisfaction serves as a mediator between system quality and perceived net benefits, thus linking technical performance to user experience and outcomes.

In HIS, user satisfaction encompasses various factors, including system reliability, user-friendly interfaces, and responsive support. For example, an intuitive HIS interface that facilitates quick data entry and retrieval contributes to higher satisfaction among healthcare providers. Studies have suggested that training and continuous technical support also play a vital role in enhancing satisfaction by empowering users to leverage system functionalities effectively (12).

However, achieving user satisfaction with outsourced HIS settings remains challenging. Vendors often struggle to align system functionalities with user needs, leading to frustration and underutilisation. Wang and Yang (9) reported that only 33% of users expressed satisfaction with outsourced systems, highlighting the need for improved vendor-user collaboration. In Malaysia, dissatisfaction with HIS often stems from integration issues and lack of user-centric design, which undermine system adoption and perceived benefits (2).

### Perceived Net Benefits

Perceived net benefits refer to the extent to which an information system (IS) contributes to the success of its users and organisation. At the individual level, these benefits include improved decision-making, enhanced productivity, and higher job satisfaction. For organisations, they encompass cost savings, increased operational efficiency, and improved service delivery (1).

In the healthcare sector, the HIS is specifically designed to reduce operating costs, optimise resource allocation, and enhance patient care. For example, integrating patient details into medication prescriptions can minimise waiting times and streamline medicine delivery processes. HIS eliminates the need for physical prescription slips by enabling electronic orders, reducing manpower requirements, and mitigating the risk of misplaced documents. Other examples include seamless clinic appointment management, immediate access to patient records, and instant availability of lab results upon completion. HIS also enhances efficiency in patient registration, appointments, ward admissions, discharges, and transfers (13). Similarly, studies have reported significant reductions in patient admission waiting times, further emphasising the potential to improve healthcare service delivery (14).

The transformative impact of HIS on organisational efficiency is well-documented. Integrated HIS has been shown to streamline clinical workflows, reduce redundant testing, and improve data accuracy, leading to significant cost savings and better patient outcomes (8). For instance, automated systems for appointment scheduling and patient tracking reduce administrative overhead, thereby freeing resources for direct patient care.

However, the realisation of these benefits is contingent on the alignment of the HIS with organisational goals and user needs. Even the most technically advanced systems may fail to deliver perceived benefits if they are not user-friendly or do not cater to the specific requirements of healthcare providers. This highlights the critical role of user satisfaction as a mediating factor. High levels of satisfaction foster trust, encourage consistent system usage and amplify perceived net benefits (1, 7).

Challenges in achieving net benefits are particularly pronounced in Malaysian public hospitals. Resource constraints, coupled with fragmented system integration, hinder HIS performance and limit its effectiveness. For example, the lack of interoperability between legacy systems and newer platforms often leads to inefficiencies and user dissatisfaction. Addressing these issues requires a dual focus on enhancing provider system quality and fostering user satisfaction to unlock the full potential of HIS and deliver tangible benefits to staff and organisations.

## Methods

### Instrument

This study employs a questionnaire to collect data, comprising four sections: respondent profiles, provider quality (including service quality, information quality, and system quality of the provider), mediating variables (user satisfaction), and the perceived net benefits of outsourcing HIS in the Ministry of Health Malaysia hospitals. The profile of respondents included age, gender, race, position, highest education level, and years of service in the current hospital of study.

The section addressing the provider’s quality, user satisfaction, and perceived net benefits is adapted from the DeLone and McLean IS success model discussed above, as it has been tested and validated in several health information systems studies in developed and developing countries (15). The 24-item questionnaire was adopted from Ojo (16), who was granted permission to use it. The questions assessing each construct were adapted from prior studies with validated scales (17–21). Each provider quality construct—system quality, information, and service quality—consists of four items. User satisfaction was measured using three items, and net benefits were measured using five items. The scale ranged from 0 (“strongly disagree”) to 10 (“strongly agree”), with the midpoint 5 labelled as “neither agree nor disagree.”

The English version of the questionnaire was translated into the local Malay language by two independent translators (a qualified linguistic expert from *Dewan Bahasa dan Pustaka* and a local bilingual researcher from the Pharmaceutical Division, Ministry of Health, Malaysia) using the forward method. The translations were then switched between them so that they could be translated back into English (backward method). A session was then held with both of them to discuss the translation to ensure that its true meaning was preserved.

The pilot test (*n* = 30) showed that the instrument used in this study had high internal consistency, with Cronbach’s alpha values of 0.880 for system quality, 0.889 for information quality, 0.866 for service quality, 0.906 for user satisfaction, and 0.903 for perceived net benefits.

### Location, Samples, and Data Collection Procedures

The study sites included six public hospitals (Hospital Sultan Ismail, Hospital Sultanah Nur Zahirah, Hospital Sultan Haji Ahmad Shah, Hospital Sultanah Bahiyah, Hospital Lahad Datu and Hospital Bintulu) in eight states: Kedah, Terengganu, Pahang, Federal Territory, Selangor, Johor, Sarawak, and Sabah. These sites were chosen based on the four different providers and systems used.

This was cross-verified using an established web-based academic tool, Daniel Soper’s sample size calculation tool for structural equation modelling (22). This calculator determines the required sample size by considering the number of observed variables (20), latent variables (5), anticipated effect size (0.3), desired probability level (0.05), and statistical power level (0.9). The recommended minimum sample size was 200. However, as in any quantitative study, withdrawals or missing data may affect the required sample size. To mitigate this, 10%–20% oversampling is recommended (23).

Based on the calculated sample size, adjustments were made to account for the response rate by dividing the required sample size by the expected response rate (1 – nonresponse rate) (24). Consequently, the final adjusted sample size was determined to be 250 samples per site. This study successfully collected actual data from 1,376 participants (Table 1).

The respondents for this study were Malaysians employed by the Ministry of Health (permanent or contract) who had worked at the selected hospitals for at least three months and regularly used the HIS in their daily work. The exclusion criteria were house officers, postgraduate students, IT staff, and staff from other hospitals who were temporarily attached or deployed to the study hospitals. These groups were excluded because they were not permanent or contract employees with consistent HIS usage.

Samples were drawn randomly from a sampling frame using the stratified random sampling technique. Each hospital was divided into three strata for equal distribution: administrative (registration counter, billing counter), clinical staff (doctors, nurses), and clinical support staff (lab technicians, radiographers, dieticians, rehabilitation therapists, pharmacists). This would reflect the total coverage of HIS function and use in the hospital.

### Statistical Analyses

No missing values were recorded for any variables in this study. The absence of missing values indicates that the data collection process was thorough and of high-quality. This ensures that the validity of the statistical analysis is not compromised by data deficiencies. Furthermore, data handling measures such as imputation or list-wise deletion were not required in this study.

This study employed structural equation modelling using Analysis of Moment Structures (AMOS) version 24 to validate the proposed research model. This decision was based on two main reasons: first, the objective of this study was to identify key predictors of intention by examining the mediating effects of provider quality (including service quality, information quality, and system quality), mediating variables (user satisfaction), and the perceived net benefits. Second, AMOS is suitable for handling large sample sizes with normally distributed data. The multivariate normality test indicated that skewness values ranged from −0.04 to +0.90 (within the acceptable range of −2 to +2), and kurtosis values ranged from −0.06 to +0.90 (within the acceptable range of −7 to +7) (25, 26). These results confirm that the data are approximately normally distributed.

The AMOS procedures followed a twostage approach, beginning with an examination of the measurement model and proceeding to the structural model. Measurement model to ensure the basic components’ reliability and validity. This involved evaluating the composite reliability (CR) and average variance extracted (AVE). The CR was required to be at least 0.70 (27), respectively, indicating adequate internal consistency. The AVE for each construct should exceed 0.50 (28), suggesting that the construct’s items explain more variance than those of the other constructs. Discriminant validity was evaluated using the Fornell–Larcker criterion, which determines whether the constructs are distinct from one another (29). Subsequently, the fitness indices met the required levels for assessment. If the fit indices were insufficient, the loading factor of each item was evaluated. Items with loadings below 0.50 were removed (26). Additionally, we estimated the goodness-of-fit indices (GOF). If the GOF did not meet the stipulated criteria, the indices were modified, or items with low factor loadings were removed.

Meanwhile, the analysis structural model consists of the *R*-squared value and coefficient (estimate *β*). The *R*-squared (*R*^2^) values indicate the proportion of variance in the dependent variables that can be explained by the independent variables. The coefficient (estimate *β*) represents the strength and direction of the relationship between the independent and dependent variables in the structural model. A two-tailed *P*-value is used to assess whether the coefficient is significantly different from zero. If the *P*-value is less than 0.05, the coefficient is considered statistically significant, meaning that there is a significant relationship between the independent and dependent variables in the model.

### Suspicious Response Pattern and Common Method Bias

Before analysing the data, the respondents’ responses must be examined to detect suspicious response patterns, often described as straight (30). Straight lining is when the respondent marks the same response for a high proportion of the questions. The results show no straight lining in the dataset. The value standard deviation is 0.4545 until 2.774, which suggests that no data were removed from the dataset. Meanwhile, Common Method Bias may be a potential concern. We used Harman’s single-factor method to assess the Common Method Bias (31). The unrotated principal component factor analysis (omitted for brevity) indicates that there is 33.1% total variance in Harman’s single-factor test, less than 50%, indicating that no single-factor loaded on all measures, which suggests that there is no Common Method Bias. Based on the results of these two methods, we confirmed that the Common Method Bias does not exist in this study.

## Result

### Demographic Profile

The demographic profile of respondents, as shown in Table 2, indicated that the respondents were predominantly female (77.6%), aged 31–40 years (51.5%), holding clinical positions (81.2%), and possessing a diploma (59.0%). Most respondents also had substantial tenure (more than 36 months), which suggests they were well-experienced with the systems being assessed. This demographic distribution provides a relevant and informed sample for evaluating healthcare information systems.

### Mean, Construct Reliability, and Validity

Table 3 presents the mean values, construct reliability, and validity analyses. The high mean scores (e.g., above 6.0 for most items) indicate positive perceptions of HIS quality, user satisfaction, and perceived benefits. Most Confirmatory Factor Analysis (CFA) loadings are strong ( > 0.70) and greater than 0.50, confirming that the items correspond well with their respective constructs. All constructs exceed the threshold for CR ( > 0.70), indicating that the items reliably measure system quality (0.846), information quality (0.935), service quality (0.969), user satisfaction (0.810), and perceived net benefits (0.883). These constructs capture more variance than the errors. Additionally, all constructs have AVE values above 0.500, namely system quality (0.586), information quality (0.782), service quality (0.888), user satisfaction (0.591), and perceived net benefits (0.656), demonstrating that the items effectively converge to represent their constructs.

Table 4 evaluates the discriminant validity using the Fornell–Larcker criterion, which determines whether the constructs are distinct from one another. The results confirm that the constructs are distinct, thus supporting the validity of the measurement model.

The constructs and items in this study are reliable and valid for assessing the intended aspects of the HIS system, indicating the robustness of the model and the integrity of the results.

### Model Fitness

Table 5 presents the results of the GOF indices for the pooled CFA model and the structural model. All GOF indices for the pooled CFA and the structural model meet or exceed their respective cut-off values. CMIN/DF (3.057 and 2.223) indicates that the model has a simple specificity. The model demonstrates excellent overall fit, with indices RMSEA (0.039 and 0.030) and RMR (0.067 and 0.067) indicating minimal error, and CFI (0.986 and 0.992), TFI (0.983 and 0.990), NFI (0.980 and 0.986), and IFI (0.986 and 0.992) showing high comparative and incremental fit. This result indicates that the pooled CFA and the structural model are well-specified and provide a robust fit to the data, confirming the reliability and validity of the measurement model and the structural relationships in the study.

### Direct Effect Relationship

**Figure 2:**
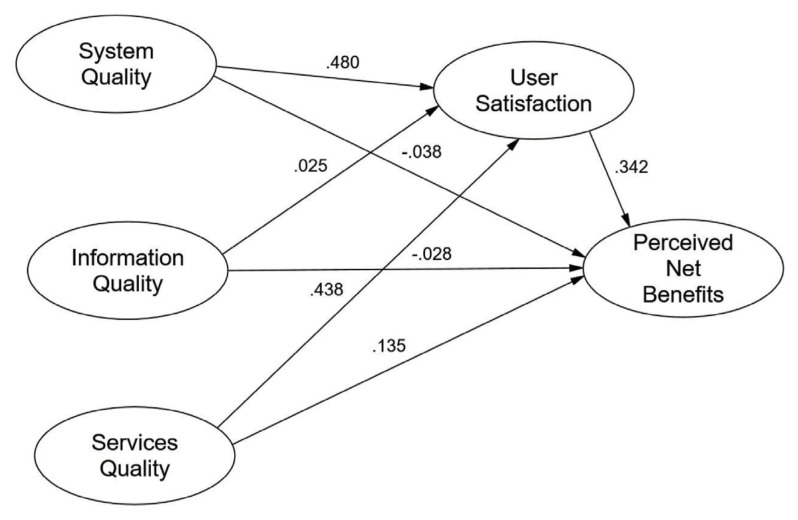
Output model

Table 6 and Figure 2 present the direct effect relationships between system quality, information quality, service quality, user satisfaction, and perceived net benefits. The *R*-squared (*R*^2^) values represent the proportion of variance explained by the independent variables in the model for each dependent variable. The *R*-squared value for user satisfaction was 0.626, indicating that 62.6% of the variance in user satisfaction can be explained by system quality, information quality, and service quality. The *R*-squared value for perceived net benefits was 0.282, indicating that 28.2% of the variance in perceived net benefits was explained by system quality, information quality, service quality, and user satisfaction.

The results indicate that the system quality of the provider has a significant (*P* < 0.001) and positive effect (*β* = 0.480) on user satisfaction, which confirms the strength and significance of this relationship, suggesting that improvements in the system quality substantially enhance user satisfaction.

However, the information quality of the provider did not have a significant direct effect (*P* = 0.232) on user satisfaction. In contrast, the service quality demonstrated a strong and highly significant (*P* < 0.001) and positive (*β* = 0.438) impact on user satisfaction. This highlights the critical role of service quality in enhancing user satisfaction.

For perceived net benefits, the system quality of the provider had an insignificant effect (*P =* 0.290), indicating no meaningful relationship. Similarly, the information quality of the provider had an insignificant effect (*P* = 0.222) on perceived net benefits. However, service quality had a significant (*P* < 0.001) and positive effect (*β* = 0.135) on perceived net benefits, underscoring the importance of high service quality in driving benefits from the system.

Lastly, user satisfaction significantly (*P* < 0.001) and positive (*β* = 0.342) influences perceived net benefits. This finding indicates that satisfied users perceive greater benefits from the system, which makes user satisfaction a key mediator between system quality dimensions and perceived benefits.

### Indirect Effects Relationship

Table 7 provides an analysis of the indirect effect relationships, examining the mediating role of user satisfaction in the relationship between system quality, information quality, service quality, and perceived net benefits.

The relationship between the provider system quality and the perceived net benefits, mediated by user satisfaction, was positive (*β* = 0.164) and significant (*P* < 0.001). This is categorised as competitive mediation, indicating that while system quality has direct and indirect effects on perceived net benefits, the indirect pathway via user satisfaction plays a prominent role.

For the relationship between provider information quality and perceived net benefits through user satisfaction, the indirect effect is positive (*β* = 0.009) but insignificant (*P* = 0.456). This suggests that information quality does not influence perceived net benefits indirectly through user satisfaction.

The final relationship, in which user satisfaction mediates the effect of provider service quality on perceived net benefits, is positive (*β* = 0.150) and significant (*P* < 0.001). These results confirm the existence of mediation, which is classified as complementary mediation. This indicates that service quality positively affects perceived net benefits directly and indirectly via user satisfaction, and the indirect pathway enhances the total effect.

## Discussion

The findings of this study underscore the significant direct and indirect relationships between system quality, service quality, user satisfaction, and perceived net benefits, providing theoretical and practical insights for system improvement and user engagement. The relationship between the provider system quality and user satisfaction indicates a positive and significant direct effect of system quality on user satisfaction. This study aligns with findings from previous studies, such as those by DeLone and McLean (1) and Chang et al. (32), which suggest that a well-designed, user-friendly, and efficient system enhances user satisfaction. This highlights the importance of continuous improvement in system performance to meet user needs. Practitioners should focus on integrating advanced technologies and streamlining system processes to improve functionality and reliability because these are pivotal in fostering user satisfaction.

The relationship between the service quality of the provider and user satisfaction indicates a significant impact of service quality on user satisfaction, which is consistent with the research by Parasuraman et al. (11), who identified service quality as a key determinant of satisfaction across industries. This indicates that timely support, professional assistance, and personalised user services are crucial for improving user satisfaction. Organisations should invest in training service personnel and employ robust feedback mechanisms to ensure that user needs are effectively addressed.

The relationship between provider information quality and user satisfaction is contrary to expectations, and information quality does not have a significant effect on user satisfaction. This finding differs from previous studies (33, 34), which found a positive link between high-quality, relevant information, and satisfaction. Likewise, a study conducted by Shih (35) indicated that information quality and user satisfaction have significant effects on HIS outsourcing. This discrepancy suggests that users in this context may prioritise system and service quality over the precision or relevance of the information provided, or the information quality may meet a baseline standard that does not significantly influence satisfaction.

Furthermore, the relationship between the service quality of the provider and the perceived net benefits indicates that it is the only significant direct driver of the perceived net benefits, emphasising its critical role in delivering user-perceived value. This study aligns with Ariyanto, Rohadi, and Lestari (34), who indicated that user satisfaction significantly affects net benefits. The insignificant effects of system and information quality on perceived net benefits suggest that these dimensions influence net benefits primarily through user satisfaction, which is a mediating factor.

The role of user satisfaction as a mediator indicates that user satisfaction plays a vital mediating role in the relationships between the system quality of the provider and perceived net benefits, as well as between service quality and perceived net benefits. These findings align with studies like those by Seddon and Kiew (36), which emphasise the importance of satisfaction in bridging system performance and user-perceived outcomes. The competitive mediation observed for system quality and complementary mediation for service quality indicate that focusing on improving user satisfaction can amplify the overall benefits of the proposed system.

However, no significant indirect relationship was observed between the information quality of the provider and the perceived net benefits through user satisfaction, suggesting that information quality alone may not substantially influence the perceived benefits in this context. Future studies should explore whether other factors, such as trust and usability, can act as mediators.

These practical implications emphasise the importance of focusing on system quality and service excellence to maximise benefits. In terms of system design and development, practitioners should focus on enhancing the functionality, reliability, and user-friendly nature of the system. Ensuring these qualities can significantly increase user satisfaction, which in turn leads to greater perceived net benefits. Regular system updates and timely bug fixes are essential to maintain high levels of system quality and prevent any negative impact on user experience.

Service excellence also plays a crucial role. Investment in service training and the development of strong customer support infrastructure can directly increase user satisfaction. When users experience consistent and efficient service, they are more likely to perceive tangible benefits from the system. Ensuring that service personnel are well-trained and responsive is key to maximising user satisfaction.

Although it remains important to reassess information quality, it may not be the most critical factor influencing the perceived net benefits in this particular context. Practitioners should focus on ensuring that the information provided meets user requirements without overemphasising it to the detriment of system and service quality. While accurate and relevant information is important, system and service improvements should be prioritised because they have a more direct impact on user satisfaction and perceived benefits.

## Conclusion

In conclusion, this study has expanded the DeLone and McLean IS success model by validating its effectiveness in the context of public hospitals in Malaysia. The findings demonstrate the model’s robustness in explaining the dynamics of system, service, and information quality as influencing user satisfaction and perceived net benefits. This study highlights the critical mediating role of user satisfaction, providing deeper insights into user interactions in healthcare settings. In addition, it offers valuable contributions to understanding how system and service quality influence user satisfaction and perceived net benefits, along with practical recommendations for improving healthcare information systems. These insights are essential for hospital administrators and policymakers to enhance strategic planning and management, ultimately improving service quality, user satisfaction, and the overall effectiveness of healthcare systems.

## Figures and Tables

**Table 1:** Actual sample size according to hospital

No.	Hospital of study	Actual sample
1.	Hospital Sultan Ismail	232
2.	Hospital Sultanah Nur Zahirah	236
3.	Hospital Sultan Haji Ahmad Shah	217
4.	Hospital Sultanah Bahiyah	226
5.	Hospital Lahad Datu	228
6.	Hospital Bintulu	237

**Table 2:** Demographic profile of the respondents

Demographic profile	*n*	%
Gender		
Male	308	22.4
Female	1,068	77.6
Age		
20–30 years old	291	21.1
31–40 years old	708	51.5
41–50 years old	307	22.3
Over 50 years old	70	5.1
Position		
Admin/Management	57	4.1
Clinical	1,117	81.2
Clinical support	202	14.7
Qualification		
SPM/STPM	39	2.8
Certificate	11	0.8
Diploma	812	59.0
Post-diploma	25	1.8
Degree/MBBS/MD	352	25.6
Postgraduate	137	10
Tenure		
3–12 months	139	10.1
12–36 months	264	19.2
More than 36 months	973	70.7

Notes: SPM = Sijil Pelajaran Malaysia (Malaysian Certificate of Education); STPM = Sijil Tinggi Persekolahan Malaysia (Malaysian Higher School Certificate); MBBS = Bachelor of Medicine, Bachelor of Surgery; MD = Doctor of Medicine

**Table 3:** Mean, construct reliability and validity

Construct/Item	Mean	CFA Loading	CR	AVE
**System qualit**y			0.846	0.586
I find the HIS easy to use.	6.04	0.985		
I find it easy to get the HIS to do what I want.	4.99	0.593		
The HIS is flexible to interact with.	5.29	0.764		
Learning to operate the HIS was easy for me.	5.40	0.621		

**Information quality**			0.935	0.782
The information generated by the HIS is correct.	6.25	0.985		
The information generated by the HIS is useful for its purpose.	6.36	0.855		
The HIS generates information in a timely manner.	6.20	0.776		
I trust the information output of the HIS.	6.33	0.909		

**Service quality**			0.969	0.888
There is adequate technical support from the system’s provider.	6.27	0.967		
The overall infrastructure in place is adequate to support the HIS.	6.08	0.915		
The HIS can be relied on to provide information as and when needed.	6.26	0.938		
The output of the HIS is complete for work processes.	6.40	0.959		

**User satisfaction**			0.810	0.591
I am satisfied with the function of the HIS.	6.54	0.903		
The HIS has eased work processes.	6.11	0.704		
I am generally satisfied using the HIS.	6.77	0.694		

**Perceived net benefits**				
The HIS will help overcome the limitations of the paper-based system.	7.85	0.733	0.883	0.656
Using the HIS will cause an improvement in patient care delivery.	7.59	0.882		
The HIS facilitates easy access to patient information.	7.92	0.843		
The HIS will enhance communication among staff.	7.33	0.696		
HIS use will cause improved decision-making.	7.34	0.821		

Notes: CFA = Confirmatory Factor Analysis; AVE = Average Variance Extracted; CR = Composite Reliability

**Table 4:** Fornell–Larcker criterion

Construct	1	2	3	4	5
User satisfaction (1)	**0.769**				
System quality (2)	0.623	**0.766**			
Information quality (3)	0.181	0.220	**0.885**		
Quality services (4)	0.706	0.431	0.148	**0.942**	
Perceived net benefits (5)	0.491	0.279	0.066	0.454	**0.810**

**Table 5:** Goodness-of-fit

Index	Cut-off value	Reference	Pool CFA	Structural model
CMIN/DF	≤ 5.00	Kline 2023	3.057	2.223
RMSEA	≤ 0.08	Hu and Bentler 1998	0.039	0.030
RMR	≤ 0.10	Bentler 1995	0.067	0.067
GFI	≥ 0.85	Awang et al. 2018	0.968	0.977
CFI	≥ 0.85	Awang et al. 2018	0.986	0.992
TFI	≥ 0.85	Awang et al. 2018	0.983	0.990
NLI	≥ 0.85	Awang et al. 2018	0.980	0.986
IFI	≥ 0.85	Awang et al. 2018	0.986	0.992

Notes: CFA = Confirmatory Factor Analysis; RMSEA = Root Mean Square Error of Approximation; RMR = Root Mean Square Residual; GFI = Goodness-of-Fit Indices; CFI = Comparative Fit Index; TFI = Tucker-Lewis fit index; NFI = Normed Fit Index; IFI = Incremental Fit Index

**Table 6:** Direct effect relationship

Relationship	Estimate (*β*)	Standard error	Critical ratio	*P*-value
Relation among System Quality and User Satisfaction	0.480	0.030	15.968	< 0.001
Relation among Information Quality and User Satisfaction	0.025	0.021	1.195	0.232
Relation among Services Quality and User Satisfaction	0.438	0.019	22.785	< 0.001
Relation among System Quality and Perceived Net Benefits	−0.038	0.036	−1.059	0.290
Relation among Information Quality and Perceived Net Benefits	−0.028	0.023	−1.222	0.222
Relation among Services Quality and Perceived Net Benefits	0.135	0.027	4.922	< 0.001
Relation among User Satisfaction and Perceived Net Benefits	0.342	0.044	7.758	< 0.001

**Table 7:** Indirect effect relationship

Relationship	Estimate (*β*)	Upper	Lower	*P*-value	Status
System Quality → User Satisfaction → Perceived Net Benefits	0.164	0.212	0.123	< 0.001	Exist mediation (competitive mediation)
Information Quality → User Satisfaction → Perceived Net Benefits	0.009	0.022	−0.003	0.456	No mediation
Services Quality → User Satisfaction → Perceived Net Benefits	0.150	0.188	0.115	< 0.001	Exist mediation (complementary mediation)
